# Can the Psychosocial Safety Climate Reduce Ill-Health Presenteeism? Evidence from Chinese Healthcare Staff under a Dual Information Processing Path Lens

**DOI:** 10.3390/ijerph17082969

**Published:** 2020-04-24

**Authors:** Beini Liu, Qiang Lu, Yue Zhao, Jing Zhan

**Affiliations:** 1 School of Business, Beijing Technology and Business University, Beijing 100048, China; 2 School of E-Business and Logistics, Beijing Technology and Business University; Beijing 100048, China; 3 School of Labor Economics, Capital University of Economics and Business; Beijing 100070, China

**Keywords:** psychosocial safety climate, perceived instrumental support, perceived emotional support, organic structure, ill-health presenteeism

## Abstract

Because of heavy workloads, non-transferable responsibilities, and shift systems, healthcare staff are prone to ill-health presenteeism. Based on social information processing theory, this study explored the influence of the psychosocial safety climate (PSC) on ill-health presenteeism. The mediating effects of perceived instrumental support and perceived emotional support and the moderating effect of organic structure in this process were observed. Using a time-lagged research design, data from 386 healthcare staff were gathered and multiple regression and bootstrapping were used to test each hypothesis. The results showed that: (1) PSC negatively relates to ill-health presenteeism. (2) Both perceived instrumental support and perceived emotional support mediate the relationship between PSC and ill-health presenteeism. The affective information processing path is more effective than the cognitive information processing path, but they do not convey a positive interaction effect on ill-health presenteeism. (3) The organic structure moderates the mediating effect of perceived emotional support but does not exert a significant moderating effect on the mediating process of perceived instrumental support. This study particularly identified PSC as a contextual antecedent of ill-health presenteeism. By combining organizational, work-related, and person-related factors, a more comprehensive theoretical framework for the understanding of ill-health presenteeism is developed, thus informing health promotion management.

## 1. Introduction

Presenteeism is generally defined as the phenomenon of attending work despite existing health problems [1,2]. In this study, presenteeism refers to the individual-centered behavior of working in ill-health conditions. Restricted by health-related diseases and pains, ill-health presenteeism places employees in a gray area between full work engagement and absence from work [3]. Ill-health presenteeism often causes employees to make more errors, have more accidents [4], and leads to lower levels of work performance and productivity because of health-related issues [3]. Therefore, ill-health presenteeism may in fact cause higher productivity losses for an organization than absence [5,6].

Based on the Confucian traditional culture of diligence, dedication, and persistence, Chinese employees are more likely to attend work even though they are ill [7,8,9]. In addition, since loyalty and reciprocity are highly respected and valued virtues within the Chinese culture, Chinese employees are more likely to partake in ill-health presenteeism to uphold a positive image and avoid sanctions related to social norms [10]. A previous study has shown that 74% of Chinese employees have engaged in work while being unhealthy [11]. In Chinese enterprises with prevailing overtime work culture, employees also show more ill-health presenteeism [10].

Effective management of ill-health presenteeism is helpful toward decreasing the risks of human resources management (HRM) with regard to: (a) productivity, (b) employee health and well-being, and (c) absence rates or patterns [12]. Several scholars have suggested that to improve the success of an organization, HRM practices need to more systematically manage ill-health presenteeism [13,14]. Therefore, this phenomenon has received extensive attention from both scholars and practitioners in the fields of organizational health, organizational management, and HRM [2].

Previous study has shown that compared with absence, ill-health presenteeism is more influenced by work-related factors [15]. Scholars have called for increased attention on the influence of less proximal work-related factors on ill-health presenteeism (e.g., organizational culture and climate) [16]. Psychosocial factors are essential in the decision-making process underlying ill-health presenteeism [17]. Although the significant influence of psychosocial factors on ill-health presenteeism has been examined, knowledge on how psychological factors affect the process mechanism of this type of presenteeism are still poorly understood [18].

The psychosocial safety climate (PSC) is a facet-specific dimension of organizational climate. It refers to shared perceptions of policies, practices, and procedures for the protection of workers’ psychological health and safety and of the practices that should be implemented to support such perceptions [19]. Previous studies have used PSC as a “job resource” and explored its relationship with psychological risks or injuries based on the job demand-resource (JD-R) theory [20] or the conservation of resources (COR) theory [21]. This study regards PSC as shared perceptions of the individual level and a kind of “social information” that can promote individual resource perception. The social information processing (SIP) theory points out that the social environment provides information that affects both the attitudes and behaviors of individuals. The goal is for individuals to better understand the working environment by processing social information, thus shaping their resulting attitudes and behaviors [22]. The SIP theory provides a suitable theoretical framework for the exploration of the internal mechanisms underlying both PSC and ill-health presenteeism.

Perceived organizational support (POS) refers to the belief of employees that the organization is concerned with their well-being and will provide necessary aid when they need to cope with stress [23]. Ill-health presenteeism, as a typical stress in the workplace, has potential risks to physical and psychological health. Therefore, POS can effectively help employees deal with this harmful and stressful situation, making it possible to unravel the mechanism between PSC and ill-health presenteeism. According to the perspective of the cognitive–affective personality system (CAPS) [24], employees will utilize two processing paths of cognition and affect when processing social information. Therefore, this study explored the intrinsic mechanism of ill-health presenteeism-related decision-making from the following two perspectives: perceived instrumental support and perceived emotional support [25].

Furthermore, scholars have proposed that the decision-making process of presenteeism should be comprehensively understood by studying key moderating roles, such as the organizational environment and its structure [16]. The SIP theory also emphasizes that the organizational situational factor will affect employees’ dependence on social information, with which they interpret their work environment and which influences their behavior [22]. Scholars have called for ill-health presenteeism to be investigated to identify the decision-making process by combining organizational, work-related, and person-related factors [2]. To address this, this study assumes the organizational structure to be an organic structure and explores the boundary conditions to assess the effectiveness of PSC.

In summary, the present study holds the basic understanding of ill-health presenteeism that focus on the individual-centered working behavior in ill-health conditions. Based on SIP theory, this study explores the theoretical model of the influence of PSC on ill-health presenteeism through perceived instrumental support and perceived emotional support from the two information-processing pathways—cognition and affect. Moreover, the situational role of organic structure in this process is examined. The results could help organizations to obtain a deeper understanding of ill-health presenteeism, promote the creation of a sustainable working environment, decrease risks related to human resources, and provide further information to promote health management.

## 2. Literature Review and Hypotheses

### 2.1. Concept Development and Theoretic Perspective of Ill-Health Presenteeism

The existing research mainly presents three definitions for presenteeism. First, European research defines presenteeism as attending work while ill [1,3,26]. Most scholars in the field of organizational management have adopted this definition [9,10,27,28]. The specific characteristic of this definition is the conceptualization of the behavior of presenteeism as the result of whether sick employees choose to go to work or not. This definition avoids the inclusion of motives and consequences of presenteeism, to avoid confusing cause and effect [29]. In addition, this definition does not obscure the possible positive impacts of presenteeism that are otherwise often neglected [2].

Second, North American research has mainly focused on productivity losses caused by attending work with health problems [11,30,31]. Here, health problems include acute minor (e.g., common cold), periodic (e.g., migraine headaches), and chronic diseases (e.g., diabetes), along with health-damaging behaviors (e.g., smoking) [2]. This defines presenteeism as the cost of health damage, while ignoring the fact that not every health problem necessarily leads to loss of productivity and ignoring the functional impact of presenteeism on future health and work [16].

Third, researchers have attempted to broaden the scope of presenteeism by considering other factors why individuals cannot fully engage in work, rather than focusing on the impact of sickness alone. For example, employees may not be able to concentrate on their work because of mental stress [32] or they may be physically present but functionally absent due to other events that distract one from full productivity (e.g., office politics) [26,33]. This definition can comprehend the behavior of employees who are neither sick nor working at the workplace, but it is still limited to productivity loss. Furthermore, this concept includes factors that are not related to sickness, which increases its generality, while decreasing its focus on health [16].

The present study assumed that the basic understanding of presenteeism should focus on the individual-centered behavior of working in ill-health conditions, which includes all types of physical and mental ill-health states. This concept should focus on the behavior and should not imply any motive or consequence. Motivation is not included in the definition because presenteeism includes both approach and avoidance motives [10], thus making it difficult to determine the specific motivations of employees. Consequences are not included in the definition because simply describing the results of presenteeism as a loss of productivity may ignore its increase in productivity compared with absence from work [26]. Moreover, research must consider that a disease is not a dichotomy. Therefore, this study does not adopt the expression of sickness presenteeism. It chooses a more inclusive description: ill-health presenteeism.

Initial research on presenteeism has mostly focused on public health and occupational medicine, and scholars have generally applied the recovery theory to explain the spiral loss phenomenon of “poor health station–presenteeism–deterioration of health” [34]. In response to the attention of scholars in the field of organization management, the JD-R theory has become widely applied when exploring the “impairment” and “motivation” caused by presenteeism to employees [28,33,35]. However, no direct empirical study has investigated the mechanism of PSC and ill-health presenteeism. As for the present study, this regards PSC as shared supporting perceptions that can promote individual resource perception. This study reviewed the literature that focused on the supporting resources at the individual level. Based on the COR theory, research has shown that leadership support can decrease productivity losses caused by presenteeism by reducing employee role conflicts [31]. Based on the organizational justice theory, scholars have suggested that leadership support and support from colleagues could reduce presenteeism [36]. However, the antecedents of social support and the types of functional support have not been explored. Based on the social exchange theory, scholars have explored the buffering effect of perceived human resource management practices on presenteeism and identified the mediating effect of turnover intention [37]. This concept focuses on the influence of administrative control rather than the psychologically shared perception on presenteeism. Based on the SOR theory, scholars have confirmed that the workplace safety climate can reduce presenteeism, of which trust forms an important bridge [38]. The workplace safety climate focuses on physical safety aspects that keep employees safe from any physical harm rather than a combination of physical and psychological health.

### 2.2. Psychosocial Safety Climate Reduces Ill-Health Presenteeism

The PSC refers to shared perceptions of policies, practices, and procedures for the protection of workers’ psychological health and safety and of practices implemented to support such perceptions [19]. Based on the SIP theory, the social environment provides a variety of information that affects the attitudes and behaviors of individuals [22]. PSC as a form of the social information existing in the organizational environment can affect these attitudes and behaviors of employees. Previous studies have confirmed that organizations with a high PSC can improve safety behaviors [39], decrease risks of adverse outcomes [40], and decrease workplace behaviors that are prone to lead to injuries [21].

First, in an organization with high PSC, senior managers establish a consensus of psychological health and safety by communicating with employees and by providing signals for employees’ desired safety behaviors [19]. PSC emphasizes the importance of mental safety and health, thus reducing unhealthy behaviors by employees that are detrimental to safety at work [41]. Previous studies have shown that a shared perception of the extent to which a team is concerned about health issues significantly decreased attendance during illness [42]. PSC is a typical mental shared perception of health concerns. It can be deduced that PSC could significantly reduce ill-health presenteeism.

Second, in an organization with high PSC, senior managers will convey information to employees that the organization is concerned about the well-being and psychological safety of its employees and that their health takes priority over production objectives [39]. This will encourage employees to reduce work behaviors that are detrimental to their safety and health and decrease risks with adverse outcomes [40]. Ill-health presenteeism, as an act of working in an unhealthy state, will lead to decreased employee productivity, an increase in the accident rate, and damages to the safety and health of employees. Therefore, it can be deduced that PSC could significantly reduce the occurrence of ill-health presenteeism.

Third, in an organization with high PSC, senior managers decrease employees’ work stress through participation and commitment, thus providing support, leading to stress prevention [40]. This decreases workplace behaviors that lead to injuries [21]. Ill-health presenteeism, as a type of behavior of working in a condition of ill-health, is a type of workplace injury behavior. This will place stress on employees and damage their physical and psychological health. Therefore, it can be deduced that PSC could significantly decrease the occurrence of ill-health presenteeism. Thus, the following hypothesis is proposed:

H1: PSC negatively relates to ill-health presenteeism.

### 2.3. Mediating Effect of Perceived Organizational Support

Based on the SIP theory, individuals can better understand the working environment by processing social information [22]. Information processing utilizes two pathways—cognitive processing and affective processing [24]. POS makes employees believe that the organization is concerned with their well-being and recognizes their organizational legitimacy [23]. It is also valued as an assurance that aid will be available from the organization when it is needed to deal with stressful situations [43]. Therefore, perceived organizational support can effectively help employees deal with situations that cause harm and stress, making it possible to understand the connection between PSC and ill-health presenteeism. POS can be grouped into two broad categories according to social support—perceived instrumental support and perceived emotional support [44]. Perceived instrumental support represents the assurance that assistance will be available for problem solving, either in the form of tangible help or in the form of information. Perceived emotional support represents the perception that one is being cared for and valued, and all communication with the source of support is full of empathy and appreciation [44].

From the perspective of instrumental support, an organization with high PSC will listen to contributions from employees and increase its participation in health and safety through procedures such as consultations with trade unions and occupational health and safety representatives [19]. This improves employees’ perception of the substantive help provided by the organization toward their health and safety. An organization with high PSC will create a safe climate by formulating policies, systems, and procedures for physiological health and safety [41]. These characteristics will increase the trust of employees in the organization and help them realize that guidance, help, and support from the organization are of instrumental value.

From the perspective of emotional support, an organization with high PSC cares about employees’ well-being and transmits the management’s commitment to employees by creating a working climate of psychological health and safety [21]. This consensus will encourage employees to form stable social relationships and enhance their emotional interaction during processes of social exchange with the organization. An organization with high PSC directs management priorities toward psychological health and safety versus productivity goals [40]. Employees feel that their health and safety are both valued and cared for, thus promoting the perception of emotional support provided by the organization. Thus, the following hypotheses are proposed:

H2a: PSC positively relates to perceived instrumental support.

H2b: PSC positively relates to perceived emotional support.

Based on the SIP theory, as a social clue in the working environment, PSC provides social information. Individuals interpret the working environment through cognitive and affective information processing paths, which further influences individual behaviors [22]. In organizations with high PSC, perceived instrumental support can increase employees’ perceptions of the practical value of health and safety assistance provided by the organization. In cases of illness, employees can receive timely functional help from the organization. For example, the organization will adjust the work-related flexibility, allow employees to work from home, arrange colleagues to share work, and further supportive behaviors, thus reducing ill-health presenteeism. Perceived instrumental support will also result in transparency of information and fairness of procedures [45]; consequently, employees will not have to worry about the impact of job uncertainty caused by absence because of illness, thus reducing ill-health presenteeism.

In organizations with high PSC, perceived emotional support can promote affective communication between employees and organizations, thus stimulating employees’ affective trust [46]. Therefore, employees will not worry about losing their jobs because of illness-related absence from work, nor will they worry that colleagues will replace their positions during this period, thus reducing ill-health presenteeism. Employees will feel comfortable talking about their difficulties and pressures with the organization [47] as a result of the emotional support they receive. Aware of the respect for and valuing of health and safety, employees will believe that the organization will support them to rest at home when their health is poor, thus decreasing ill-health presenteeism. Thus, the following hypotheses are proposed:

H3a: Perceived instrumental support mediates the relationship between PSC and ill-health presenteeism.

H3b: Perceived emotional support mediates the relationship between PSC and ill-health presenteeism.

### 2.4. Moderating Effect of Organic Structure

The SIP theory emphasizes that the organizational situational factor will affect the employees’ dependence on social information in interpreting their work environment, consequently influencing their behavior [22]. As an important organizational situational factor, organizational structure is a trigger that stimulates individual cognitive–affective units [24]. Organizational structure is structural system that has formed in response to the relationships between the members of the organization [48]. Organic structure refers to an organization that does not use permanent fixed positions and departments with strictly defined functional boundaries [49]. As long as it is convenient for the employers to achieve specific work objectives, such organizations can be free from the constraints of formal rules and regulations [50].

Based on the SIP theory, when the workplace environment is highly uncertain and ambiguous, individuals rely more on clues provided by social information in interpreting the working environment and adjust their attitudes and behaviors accordingly [22]. Therefore, the uncertainty induced by an organic organizational structure will make employees more dependent on the processing of social information transmitted by the PSC, thus enhancing their perceived support and decreasing ill-health presenteeism.

From the perspective of perceived instrumental support, an organic structure leads to a more flexible and adaptable work arrangement [51]. Organizations will provide a variety of work assistance for ill-health employees. Through flexible work arrangements and adjustments, interaction and cooperation are promoted, meaning employees can easily find a replacement when they are ill, thus reducing ill-health presenteeism. Furthermore, an organic structure is highly decentralized and few standardized rules and regulations are applied [52]. Organizations do not need to be constrained by strict rules and regulations. They can flexibly help employees in poor physical condition to arrange more suitable work programs, so that their work responsibilities can be fulfilled. Therefore, an organic structure will strengthen the effect of PSC in reducing ill-health presenteeism through perceived instrumental support.

From the perspective of perceived instrumental support, an organic structure emphasizes that communication between managers and employees is no longer top-down, but also horizontal and even bottom-up [50]. Open communication will enable employees to feel the concern and respect that the organization has for them, thus enhancing the role of PSC toward promoting high-quality exchange relationships. An organic organizational structure adopts a decentralized structure, thus allowing employees to participate in organizational decisions [53]. The organization will listen to and adopt opinions on health and safety from employees. Therefore, the concern felt by the organization about the employees’ health and well-being will be more apparent. Thus, employees will believe that when they are sick, the organization will support them and encourage them to rest at home instead of continuing to work despite their poor health. Therefore, an organic structure will strengthen the effect of PSC by reducing ill-health presenteeism through perceived emotional support. Thus, the following hypotheses are proposed:

H4a: An organic structure moderates the mediating effect of PSC by reducing ill-health presenteeism through perceived instrumental support, such that the relationship is strengthened when the level of organic structure is higher (versus lower).

H4b: An organic structure moderates the mediating effect of PSC by reducing ill-health presenteeism through perceived emotional support, such that the relationship is strengthened when the level of organic structure is higher (versus lower).

**Figure 1:**
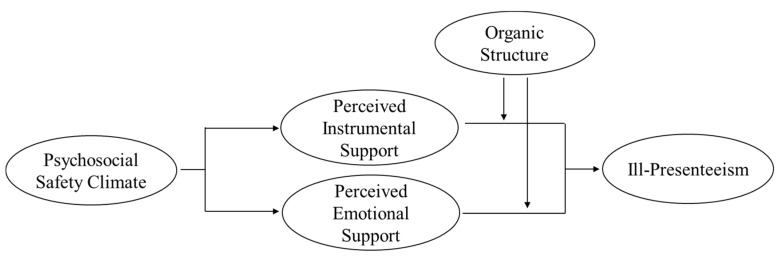
Theoretical model.

Figure 1 shows the developed theoretical model.

### 2.5. Ethical Statement

Ethical review and approval was not required for the study on human participants in accordance with the local legislation and institutional requirements. Written informed consent from the participants was not required to participate in this study in accordance with the national legislation and the institutional requirements. 

## 3. Methodology

### 3.1. Measures

All variables were obtained or adapted from previously validated instruments and were appropriately refined according to the background of the present study and the specific Chinese context. Two researchers independently translated the English scale into Chinese. The resulting Chinese questionnaire was then subjected to a back-translation procedure by two independent researchers to ensure the accuracy and consistency of the translation.

PSC was measured with the 12-item scale developed by Hall et al. [39]. An example item is, “Psychological well-being of staff is a priority for this organization”.

Perceived instrumental support and perceived emotional support sections employed subscales from the family supportive supervisor behaviors (FSSB) scale developed by Hammer et al. [25], using three-item and four-item scales, respectively. The scales were revised according to the context of ill-health presenteeism. An example item of perceived instrumental support is, “I can depend on my organization to help me support with scheduling health conflicts if I need it”. An example item of perceived emotional support is, “My organization takes the time to learn about my personal health issues”.

Organic structure was measured with a four-item scale adopted from Aryee et al. [50] and Slevin and Covin [54]. An example item is, “A strong emphasis on getting things done, even if it means disregarding formal procedures”.

Ill-health presenteeism was measured with a two-item scale developed by Demerouti et al. [27] and Aronsson et al. [55]. An example item is, “Although I feel sick, I still go to work”, which was evaluated over the past six months. Lu et al. [10] applied this scale to the Chinese context. In reference to the research results of previous scholars, gender, age, marital status, education, tenure, and employment type were used as control variables [28,31,56].

### 3.2. Sampling

This study used a time-lagged research design for data collection to decrease common method bias [57]. Questionnaires were distributed to frontline healthcare staff from public hospitals all over China. The survey lasted for six months, which is consistent with the recall period required by ill-health presenteeism measurement methods [10,27,55]. The survey was divided into three time points for data collection. To ensure correct tracking of matching questionnaires, the last four digits of the respondents’ mobile phone numbers were used as matching codes. At the first time point, PSC, organic structure, health condition, and control variables were measured. A total of 636 questionnaires were distributed, 609 were recovered, and 568 were identified as valid. According to previous studies, the following types of questionnaires were excluded from the data set: (a) healthcare staff without a record of illness over the past year, because such a low record of illness indicates that these employees do not need to consider ill-health presenteeism; (b) healthcare staff with a record of illness in the past year, because this indicates that these people are vulnerable to illness and may thus cause a causal cycle [58]. According to these criteria, 73 questionnaires were excluded and 495 valid questionnaires were entered into the second round. The second period of data collection commenced after three months and measured both the perceived instrumental support and the perceived emotional support. A total of 442 questionnaires were collected, 421 of which were valid. The last period of data collection commenced three months after the second period and measured ill-health presenteeism. A total of 398 questionnaires were collected, 386 of which were valid. This constitutes the final effective response rate of 60.7%. In this sample of healthcare staff, 70.8% were females, the average age was 35.74 years, 71.8% were married, 58.2% had a bachelor’s degree or below, the average tenure was 7.97 years, and 71.3% had permanent contracts.

### 3.3. Nonresponse Bias and Common Method Bias Tests

Before conducting formal data analysis and testing of hypotheses, nonresponse bias and common method bias were examined. This study mainly used SPSS 22.0 (IBM Corp., Armonk, NY, USA) and LISREL 8.80 (Scientific Software International, Inc., Lincolnwood, IL, USA) for data analysis and hypothesis testing. First, analysis of variance (ANOVA) was adopted to compare the mean differences between early and late respondents. This was used to test the nonresponse bias based on control variables. The results showed no significant mean differences (*p* > 0.1), thus indicating that this study had no problem with nonresponse bias.

Second, common method bias is an artificial covariant between both predicted variables and valid standard variables, which is typically caused by the same data collection condition. Harman’s single factor test is widely used by scholars because of its simplicity and ease of use [57]. Factor analysis showed that all items could be loaded into five factors. The total explanation variation degree of these extracted five factors was 72.876%, 23.759% of which was the first factor explanation variation degree. No one factor explanation was identified for most variations, thus indicating that this study does not have a severe common method bias problem.

## 4. Data Analysis and Results

### 4.1. Reliability and Validity

As shown in Table 1, the Cronbach’s α values of each construct were found to exceed the cutoff value of 0.70. Composite reliability (CR) ranged from 0.774 to 0.994, exceeding the recommended threshold of 0.7. All constructs in this study achieved good reliability.

Regarding the validity of each construct, firstly all scales used in this study originated from mature studies, the validity of which had been proven by previous empirical studies, thus ensuring that the scale of this study achieved good content validity. Secondly, as shown in Table 1, the average variance extraction (AVE) of each construct exceeded 0.5. As shown in Table 2, the results of confirmatory factor analysis showed that the model achieved good fit, which also showed that the scale achieved good convergent validity. Finally, the square root of each construct AVE exceeded the correlation coefficient between this construct and other constructs, thus indicating that the measurement scale in this study achieved good discriminate validity.

### 4.2. Hypotheses Testing

To test the effects of the PSC on ill-health presenteeism and the mediating roles of both perceived instrumental support and perceived emotional support, this study constructed six regression models. The results of collinearity statistical analysis showed that the tolerance of each model exceeded the cutoff value of 0.1 and the VIF of each model was below the cutoff value of 10. These results suggest that a collinearity problem does not exist. Moreover, Table 3 shows that the Durbin–Watson statistic of each model was approximately 2, indicating that the regression equation had no serial autocorrelation.

Direct effect of PSC on ill-health presenteeism. As shown in Table 3, the results of M1 showed that the PSC exerts a significant negative effect on ill-health presenteeism (β = −0.081, *p* < 0.05). Thus, H1 is supported.

Mediating effects of perceived instrumental support and perceived emotional support. With regard to the mediating effect of perceived instrumental support, as shown in Table 3, the results of M1 indicate that PSC exerts a significant negative effect on ill-health presenteeism (β = −0.081, *p* < 0.05). The results of M2 show that PSC exerts a significant positive effect on perceived instrumental support (β = 0.067, *p* < 0.05), which supports H2a. The results of model 4 show that perceived instrumental support has a significant negative effect on ill-health presenteeism (β = −0.053, *p* < 0.05). When perceived instrumental support is introduced as a mediator, compared with M1, the effect of PSC on ill-health presenteeism becomes significantly smaller (β = −0.078, *p* < 0.05). This indicates that perceived instrumental support plays a partial mediating role between PSC and ill-health presenteeism. Thus, H3a is supported.

Regarding the mediating effect of perceived emotional support, as shown in Table 3, the results of M1 indicate that the PSC has a significant negative effect on ill-health presenteeism (β = −0.081, *p* < 0.05). The results of M3 show that the PSC has a significant positive effect on perceived emotional support (β = 0.093, *p* < 0.01), which supports H2b. The results of Model 5 show that perceived emotional support has a significant negative effect on ill-health presenteeism (β = −0.149, *p* < 0.001). When perceived emotional support is introduced as mediator, compared with M1, the effect of the PSC on ill-health presenteeism becomes significantly smaller (β = −0.067, *p* < 0.05). This indicates that perceived emotional support plays a partial mediating role between the PSC and ill-health presenteeism. Thus, H3b is supported.

This study used bootstrapping to further verify the mediating roles of perceived instrumental support and perceived emotional support. As shown in Table 4, the indirect effect of the PSC on ill-health presenteeism through perceived instrumental support is -0.004, the standard error is 0.004, and the confidence interval is [−0.015, −0.001]. After controlling the mediating variable of perceived instrumental support, the direct effect of the PSC on ill-health presenteeism is -0.083, the standard error is 0.036, and the confidence interval is [−0.155, −0.012]. Since zero is not included in the confidence intervals of these effects, perceived instrumental support plays a significant mediating role between the PSC and ill-health presenteeism. Therefore, H3a is verified again. Similarly, perceived emotional support also has a significant mediating role between the PSC and ill-health presenteeism, and H3b is verified again. In addition, in the two mediating effect paths, because the total effect of the PSC on ill-health presenteeism is identical, perceived emotional support (with an indirect effect of −0.015) has a stronger mediating effect between the PSC and ill-health presenteeism compared with perceived instrumental support (with an indirect effect of −0.004).

In addition, this study further explored the interactive effect of perceived instrumental support and perceived emotional support on ill-health presenteeism. As shown in Table 3, when the interaction between perceived instrumental support and perceived emotional support is introduced, the results of M6 indicate that the interaction exerts no significant effect on ill-health presenteeism. This shows that perceived instrumental support and perceived emotional support cannot promote each other. In other words, the effects of perceived instrumental support and perceived emotional support on ill-health presenteeism are not mutually reinforcing and are independent of each other.

Moderated mediation effect of organic structure. This study also used bootstrapping to verify the moderating effect of an organic organizational structure on the path of the PSC influencing ill-health presenteeism through perceived instrumental support and perceived emotional support. The results of these conditional indirect effects of the moderate variable organic structure under different values are shown in Table 4. Bootstrapping is performed 5000 times.

Regarding the mediator of perceived instrumental support, the conditional indirect effect analysis in the left half of Table 5 indicates that for organizations with low organic structure, the indirect effects of the PSC on ill-health presenteeism through perceived instrumental support is -0.003, with a standard error of 0.005, and a confidence interval of [−0.018, 0.004]. For organizations with intermediate organic structure, the indirect effect of the PSC on ill-health presenteeism through perceived instrumental support is −0.002, with a standard error of 0.003, and a confidence interval of [−0.012, 0.003]. For organizations with highly organic structure, the indirect effect of the PSC on ill-health presenteeism through perceived instrumental support is −0.002, with a standard error of 0.004, and a confidence interval of [−0.009, 0.006]. Since the above confidence intervals contains zero, the moderating effect of the organic structure on the path of the PSC influencing ill-health presenteeism through perceived instrumental support is not significant. Thus, H4a is not supported.

Regarding the mediator of perceived emotional support, the conditional indirect effect analysis in the left half of Table 5 indicates that when an organization has a low organic structure, the indirect effect of the PSC on ill-health presenteeism through perceived emotional support is -0.014, with a standard error of 0.008, and a confidence interval of [−0.035, −0.002]. For organizations with an intermediate organic structure, the indirect effect of the PSC on ill-health presenteeism through perceived emotional support is −0.012, with a standard error of 0.007, and a confidence interval of [−0.029, −0.002]. For organizations with a highly organic structure, the indirect effect of the PSC on ill-health presenteeism through perceived emotional support is −0.010, with a standard error of 0.007, and a confidence interval of [−0.030, −0.001]. Since none of the above confidence intervals contain zero, regardless of whether the organic structure is low, mean, or high, the indirect effect of the PSC on ill-health presenteeism is significant through perceived emotional support.

Moreover, the right half of Table 5 reports the index of the moderated mediation effect. The data show that the indicator of organic structure that moderates the indirect relationship between the PSC and ill-health presenteeism through perceived emotional support is 0.002, with a standard error of 0.004, and a confidence interval of [0.005, 0.012]. Since the confidence interval of the moderating effect of organic structure on the indirect relationship between the PSC and ill-health presenteeism through perceived emotional support does not contain zero, the moderated mediation effect is significant. Thus, H4b is supported. The data shows that an organic organizational structure exerts a positive moderating effect on the indirect relationship between the PSC and ill-health presenteeism through perceived emotional support. This indicates that with increasing strength of the organic structure applied by an organization, the mediating effect of the PSC influencing ill-health presenteeism through perceived emotional support will be enhanced.

## 5. Discussion

### 5.1. Theoretical Contributions

PSC reduces ill-health presenteeism. First, previous studies have treated PSC as a “resource” and explored its relationship with psychological risks or injuries either based on the JD-R theory [20] or the COR theory [21]. This study regards PSC as a type of “social information”, which adds to the PSC literature in general based on the SIP theory. Second, the current research considered PSC as a lead indicator of psychological risks and harm [21,59]. Little research has focused on the process of its impact on the combination of physical and psychological health, e.g., in the context of ill-health presenteeism. The present paper broadens the knowledge associated with the outcomes of the PSC. Third, although existing research investigated the role of a supportive working environment toward reducing presenteeism [31,36], the antecedents of social support and the types of functional support have not been fully explored. This study enriches the research on the PSC as a contextual antecedent of ill-health presenteeism in particular.

Perceived instrumental support and perceived emotional support mediate the relationship between PSC and ill-health presenteeism. First, previous research on the PSC mechanism has considered either work-related mechanisms such as job demands [59] or psychologically related mechanisms such as psychological needed thwarting [60]; few studies have integrated cognitive and affective pathways. Combining the SIP theory and the CAPS perspective, this study distinguished the types of functional support and considered cognitive and affective information processing paths, thus enriching PSC literature on the mechanism of ill-health presenteeism. Second, perceived emotional support (with an indirect effect of −0.015) reduces ill-health presenteeism more than perceived instrumental support (with an indirect effect of −0.004). This conclusion is consistent with previous studies [61,62]. Such emotional support plays an overwhelming role in predicting positive behavior and well-being [61], and recipients prefer and usually benefit more from emotional concern [62]. Third, in contrast to previous research that indicated that emotional and instrumental support can promote each other [45], this study clarified the relative independence of cognitive and affective pathways under the context of ill-health presenteeism. Perceived instrumental support and perceived emotional support cannot reinforce each other. Previous studies have also shown that these two processing paths play different roles [61] and exert ambiguous interaction effects in cognitive-oriented goals [63]. The conclusion of this study also confirms that the interaction between both processing paths should be assessed with caution.

Organic structure is a situational factor that improves the effectiveness of PSC. First, it has been proposed that presenteeism should be understood by exploring the key moderating roles of organizational structure [16]. By introducing organic structure, the present study has expanded the existing knowledge of boundary conditions under which PSC can be more or less effective. Second, the present study integrated organizational, work-related, and person-related factors into one consistent research framework, thus responding to the research appeal of previous scholars [2] while also providing a more comprehensive view of the ill-health presenteeism decision-making process. Third, an organic organizational structure moderates the mediating effect of perceived emotional support. However, it exerts no significant moderating effect on the mediating process of perceived instrumental support. This may be because perceived emotional support is based on frequent interactions that makes employees feel valued and cared for. An organic structure can promote communication and decision-making participation, thus affecting the mediation path of perceived emotional support. However, perceived instrumental support represents the functional and practical judgment of employees of the psychological safety and health assistance provided by the organization. Independent of changes of the organizational structure, this objective judgment based on rational cognition will not be affected.

### 5.2. Managerial Implications

The presented research findings enrich the understanding of how PSC works in healthcare. The conclusions have the following practical significance for healthcare organizations:

Build a high-level PSC to reduce ill-health presenteeism of healthcare staff. First, based on PSC, “minimally anxiety-triggering workplaces” [64] should be designed and a working environment should be created that promotes psychological health and safety in healthcare staff. Second, organizational and job-design practices in healthcare institutions should be developed that better value the psychological health of workers. Third, senior management involvement in workplace health and safety should be improved. Transparency around decisions at the top level and potential flow-on effects of workplace safety should be exhibited [65].

Promote instrumental support and emotional support perception of healthcare staff. First, senior managers should improve and ensure the frequency of sufficient contact with frontline staff to mitigate psychosocial risks. The focus for communication should be to convey a positive attitude to staff to support workplace health and safety and ensure that they feel valued and supported. Second, an appraisal system emphasizing dimensional performance should be designed and the quality of caring instead of attendance frequency should be emphasized [66]. The workload should be adjusted and colleagues’ schedules should be arranged to share work and decrease the demanding requirements of attendance. Third, perceived emotional support can reduce ill-health presenteeism more effectively. Therefore, healthcare institutions should promote communication with staff through multiple channels. Keeping staff in the loop and helping them to understand the thought processes behind major decisions related to health and safety will help build a fair and transparent process.

Promoting the effects of health management through an organic structure. First, a strong emphasis should be placed on getting things done, even if this means disregarding formal procedures [51]. Informal cooperation should be encouraged between healthcare staff so that they can share work flexibly when they are sick. Second, managers’ operating styles should be allowed to range widely. Managers should translate PSC into action through various means, so that healthcare staff perceive management values and actions as being congruent. This integrity will serve to improve worker psychological health [41]. Third, although emotional support can reduce ill-health presenteeism more effectively, it is affected by the contingency of the organizational structure. The mediating effect of the instrumental support on ill-health presenteeism is relatively stable. Because both paths have no promoting effect, healthcare institutions can choose to use them independently according to the situation at hand.

### 5.3. Limitations and Future Research

As with all studies, this research has a number of limitations. First, while efforts were made to ensure that the utilized healthcare staff sample included a mixture of nursing, medical, allied health, and administrative staff, the findings may be limited to public sector healthcare professionals and those working in large hospitals as opposed to private practices. Future research should expand the sample source, verify the research conclusions in a wider sample range, and improve the universality of the presented research. Second, this study regards PSC as a perceived resource at the individual level. Future research can explore and enrich the theoretical model of ill-health presenteeism from different levels, such as by designing cross-layer research at the organizational level. Moreover, this study showed that both perceived instrumental support and perceived emotional support exert partial mediating roles between PSC and ill-health presenteeism. Future studies should introduce other mediation variables, thus enriching the research model and further expanding the research on the internal mechanism with which PSC reduces presenteeism. Third, the research conclusions of the interaction between instrumental support and emotional support are inconsistent [45,61,63]. In the context of the present study, this has no reinforcing effect and means that the presented interpretations of the findings should be understood with caution and that future research is warranted. Future studies should employ quasi-replication designs to verify the applicability of the obtained results.

## 6. Conclusions

Based on the SIP theory, this study confirmed that the PSC could be regarded as social information influencing interpretation of the working environment, thus reducing ill-health presenteeism among healthcare staff. Two information processing paths were identified—cognitive processing (perceived instrumental support) and affective processing (perceived emotional support)—with an organic organizational structure being an important situational factor. An affective processing path can reduce ill-health presenteeism more effectively, but its effectiveness is influenced by the organic structure of the organization. The two paths do not promote each other; therefore, they can be used independently according to the actual situation. In general, senior managers of healthcare institutions can reduce ill-health presenteeism by building a high-level PSC, promoting support perceptions, and adjusting the organizational structure.

## Figures and Tables

**Table 1:** Reliability test results for each construct.

Construct	Cronbach’s α	CR	AVE
Psychosocial Safety Climate	0.880	0.918	0.737
Perceived Instrumental Support	0.733	0.847	0.654
Perceived Emotional Support	0.715	0.774	0.536
Organic Structure	0.776	0.899	0.817
Ill-Health Presenteeism	0.988	0.994	0.988

CR: Composite reliability; AVE: average variance extraction.

**Table 2:** Correlation coefficients between each construct.

Construct	1	2	3	4	5	6	7	8	9	10	11
Gender	--										
Age	−0.089 ^**^	--									
Marriage	−0.046	0.479 ^**^	--								
Education	−0.185 ^**^	0.023	0.101 ^**^	--							
Tenure	−0.049	0.721 ^**^	0.620 ^**^	−0.076 ^*^	--						
Employment Type	0.043	−0.409 ^**^	−0.388 ^**^	−0.143 ^**^	−0.490 ^**^	--					
Psychosocial Safety Climate	0.002	0.156 ^**^	0.091 ^**^	0.022	0.137 ^**^	−0.119 ^**^	0.858				
Perceived Instrumental Support	−0.027	0.136 ^**^	0.074 ^*^	0.090 ^**^	0.031	−0.034	0.086 ^*^	0.809			
Perceived Emotional Support	0.014	0.040	0.036	0.003	0.014	0.000	0.098 ^**^	0.402 ^**^	0.732		
Organic Structure	0.095 ^**^	−0.113 ^**^	0.004	0.033	−0.032	0.007	−0.050	−0.257 ^**^	−0.244 ^**^	0.904	
Ill-Health Presenteeism	0.086 ^*^	−0.069 ^*^	0.043	0.090^**^	0.017	−0.046	−0.081 ^*^	−0.064	−0.155 ^**^	0.174 ^**^	0.994
*χ^2^* = 342.103, *df* = 157, *χ^2^*/*df* = 2.179, RMSEA = 0.064, RMSR = 0.064, CFI = 0.97, IFI = 0.97, NFI = 0.95, NNFI = 0.97											

Note: * *p* < 0.05; ** *p* < 0.01; the diagonal numbers formatted in bold show the square root of AVE. RMSEA = root-mean-square error of approximation, RMSR = root-mean-square residual, CFI = comparative fit index, IFI = incremental fit index, NFI = normed fit index, NNFI = non-normed fit index.

**Table 3:** Test results of the direct and mediating effects.

Variable	Ill-Health Presenteeism	Perceived Instrumental Support	Perceived Emotional Support	Ill-Health Presenteeism	Ill-Health Presenteeism	Ill-Health Presenteeism
	M1	M2	M3	M4	M5	M6
Gender	0.097 ^**^	−0.012	0.011	0.096 ^**^	0.098 ^**^	0.097 ^**^
Age	−0.166^***^	0.256 ^***^	0.088 ^*^	−0.152 ^**^	−0.152 ^**^	−0.154 ^**^
Marriage	0.037	0.050	0.029	0.040	0.041	0.041
Education	0.115^**^	0.062 ^*^	−0.007	0.118 ^**^	0.114 ^**^	0.112 ^**^
Tenure	0.108 ^*^	−0.142 ^*^	−0.031	0.101 ^*^	0.104 ^*^	0.104 ^*^
Employment Type	−0.043	0.062	0.066	−0.039	−0.033	−0.033
Psychosocial Safety Climate (PSC)	−0.081^*^	0.067 ^*^	0.093 ^**^	−0.078 ^*^	−0.067 ^*^	−0.068 ^*^
Perceived Instrumental Support (PIS)				−0.053 ^*^		0.001
Perceived Emotional Support (PES)					-0.149^***^	−0.151 ^***^
PIS*PES						-0.019
R^2^	0.040	0.050	0.017	0.043	0.062	0.063
*F*	5.188 ^***^	6.481 ^***^	2.076 ^*^	4.846 ^***^	7.150 ^***^	5.742 ^***^
DW	1.993	2.051	2.002	1.994	2.005	2.003

Notes: ^*^ *p* < 0.05; ^**^ *p* < 0.01; ^***^ *p* < 0.001. DW = Durbin-Watson

**Table 4:** Bootstrapping tests for the mediating effect.

Mediator	Effect	Effect Size	Standard Error	95% Confidence Interval	
				Minimum	Maximum
Perceived Instrumental Support	Indirect effect	−0.004	0.004	−0.015	−0.001
	Direct effect	−0.083	0.036	−0.155	−0.012
	Total effect	−0.087	0.036	−0.158	−0.016
Perceived Emotional Support	Indirect effect	−0.015	0.007	−0.033	−0.004
	Direct effect	−0.072	0.036	−0.143	−0.002
	Total effect	−0.087	0.036	−0.158	0.016

**Table 5:** Bootstrapping tests for mediated moderation effect.

Mediator	Effect					Effect Size			
	Moderator	Effect	Standard Error	95% Confidence Interval		Index	Standard Error	95% Confidence Interval	
				Minimum	Maximum			Minimum	Maximum
Perceived Instrumental Support	Low	−0.003	0.005	−0.018	0.004	0.002	0.003	−0.003	0.010
	Mean	−0.002	0.003	−0.012	0.003				
	High	−0.002	0.004	−0.009	0.006				
Perceived Emotional Support	Low	−0.014	0.008	−0.035	−0.002	0.002	0.004	0.005	0.012
	Mean	−0.012	0.007	−0.029	−0.002				
	High	−0.010	0.007	−0.030	−0.001				

Notes: The low and high values of the mediator variable are one standard deviation lower than and higher than the mean value of the mediator variable (organic structure), respectively, and the median value is the mean value of the mediator variable (organic structure).
